# GNSS land subsidence observations along the northern coastline of Java, Indonesia

**DOI:** 10.1038/s41597-023-02274-0

**Published:** 2023-07-01

**Authors:** Susilo Susilo, Rino Salman, Wawan Hermawan, Risna Widyaningrum, Sidik Tri Wibowo, Yustisi Ardhitasari Lumban-Gaol, Irwan Meilano, Sang-Ho Yun

**Affiliations:** 1 National Agency for Research and Innovation (BRIN), Jakarta, Indonesia; 2 Earth Observatory of Singapore, Nanyang Technological University, Singapore, Singapore; 3 Center for Groundwater and Environmental Geology, Geological Agency, Bandung, Indonesia; 4 Geospatial Information Agency (BIG), Cibinong, Indonesia; 5 Faculty of Earth Sciences and Technology, Institute of Technology Bandung (ITB), Bandung, Indonesia; 6 Asian School of the Environment, Nanyang Technological University, Singapore, Singapore; 7 School of Electrical and Electronic Engineering, Nanyang Technological University, Singapore, Singapore

**Keywords:** Natural hazards, Solid Earth sciences

## Abstract

Land subsidence in cities along the northern coastline of Java has been at a worrying level. Monitoring efforts using geodetic data reveal that Jakarta, Pekalongan, Semarang, and Demak subside at least ~9x faster than the present-day rate of global sea level rise, which affects the cities’ future urban viability. In this study, we publish a time series of the precise 3D displacements observed by twenty continuous Global Navigation Satellite System (GNSS) stations between 2010 and 2021. These are the first open-to-the-public and rigorously processed GNSS datasets that are useful for accurately quantifying land subsidence in the densely populated sinking cities in Java. The data also provides a way to tie other geodetic observations, such as Interferometric Synthetic Aperture Radar (InSAR), to a global reference frame in an attempt to build worldwide observations of coastal land subsidence.

## Background & Summary

**Fig. 1:**
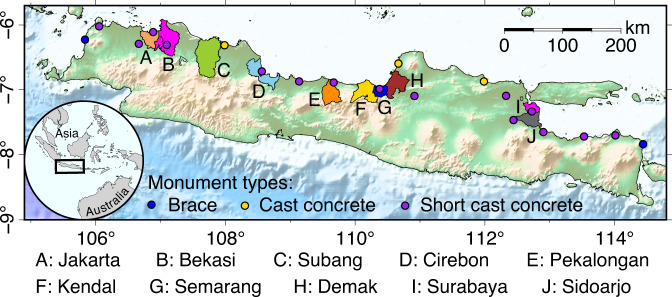
Administrative boundaries of coastal cities along the northern coastline regions of Java that are known to experience land subsidence^1–18^.

The northern coastline regions of Java have been soliciting the attention of many studies because a large portion of land in at least ten cities is subsiding^1–18^ (Fig. 1). The subsiding land has been triggered by a wide range of natural and anthropogenic activities, such as the compaction of sediments in Pekalongan, Semarang, and Demak^19,20^, gas extraction in Sidoarjo^16^, and structural loadings in Jakarta^1^. In addition, excessive groundwater extraction is the most significant triggering factor due to the increasing demand and need for residential and industrial water supply^2,16,21–24^. In these cities, the impacts of land subsidence such as widespread coastal inundation and structural damage to buildings, have been significantly reducing the quality of the living environment^1,2,5,14,17,20,25–27^. In Jakarta, the capital city of Indonesia, land subsidence is so severely affecting the city’s future urban viability^28^ that government authorities are planning to move the capital to Borneo^29^.

Monitoring efforts to study the spatial extent of land subsidence and its rates in these cities have been continuously made using land-based and space-borne techniques^1–4,6–11,15,16,27,30^. Out of the ten cities, land subsidence in Jakarta and Semarang has been the most intensively studied with a long monitoring history. In Jakarta, levelling surveys and campaign GNSS measurements between 1982 and 2010 estimate that the rates are from 1 to 28 cm/year^1^. A recent study using Sentinel-1 InSAR data between 2014 and 2020 estimates that the rates are from 1 to ~11 cm/year^7,8^. Different rates between the past and recent monitoring efforts have also been observed in Semarang: GNSS measurements between 1999 and 2011 estimate that the rates are from 14 to 19 cm/year^2^, while a recent study using Sentinel-1 InSAR data between 2015 and 2020 reveals that the rates are from 2 to 3 cm/year^8^.

Besides Jakarta and Semarang, land subsidence monitoring efforts using InSAR data in the remaining cities have been rapidly growing since 2013^3,4,6,9,11,16,31–33^ thanks to the availability of open access SAR data from the Copernicus Sentinel-1 satellites operated by the European Space Agency. On the contrary, since 2013, the monitoring efforts using campaign GNSS measurements have been lacking; the latest measurements were in 2010 for Jakarta^1^, in 2017 for Semarang^12^, and in 2018 for Demak^10^. Even worse, no study reported GNSS-based monitoring efforts in other cities that are known to experience land subsidence, such as Bekasi, Subang, Pekalongan, and Surabaya. This lack of GNSS-based monitoring efforts is worrying because the GNSS data is still needed for several reasons.

First, although InSAR observations provide all-weather and day-night monitoring capacity at high spatial coverage and resolution^34,35^, InSAR accuracy may still be degraded by various noise sources such as atmospheric phase delays, satellite orbit uncertainty, and unwrapping errors^36,37^. The degraded accuracy may mislead the interpretation of the subsidence rate. Incorporating independent data from GNSS measurements can help mitigate the false interpretation^38,39^. Second, InSAR velocity maps are relative to a reference point within the SAR data footprints. In areas where GNSS data is not available, a common approach to select a reference point is by assuming a certain area to be stable. However, this approach is subjective and may result in varying InSAR velocity maps across different studies. For example, Tay *et al*.^7^ showed that a location on the northern coastline of Jakarta subsides ~7x faster than that reported by Wu *et al*.^8^. One possible explanation for this discrepancy is the use of different reference points. Therefore, GNSS data is necessary to provide a priori information for selecting a stable reference point. Third, InSAR velocity maps are 1D measurements of surface deformations in the radar line-of-sight direction of SAR satellites^34,35^. In the case of land subsidence monitoring where vertical motions are of interest, other data sets such as GNSS observations are needed to isolate the vertical motions precisely (e.g.^40–43^). Fourth, Shirzaei *et al*.^44^ suggest the need for incorporating geocentric global reference frame vertical land motion (VLM) into global mean sea level (GMSL) studies. Geocentric is the natural for a global frame. Therefore, GMSL studies relative to this frame will allow us to determine whether a given location is rising or falling relative to the centre of the Earth. The InSAR-based VLM measurements are ideal for this purpose because InSAR data provide global coverage observations. However, the main challenge is that InSAR results are provided in a local reference frame. Thus, establishing worldwide InSAR-based VLM measurements needs GNSS data to tie the VLM measurements into a global reference frame^44^. In this study, we publish a time series of 3D displacements observed at twenty continuous GNSS stations between 2010 and 2021 along the northern coastline regions of Java (Fig. 1). The data may potentially be used for all the purposes mentioned above.

### Observation specifications

**Fig. 2:**
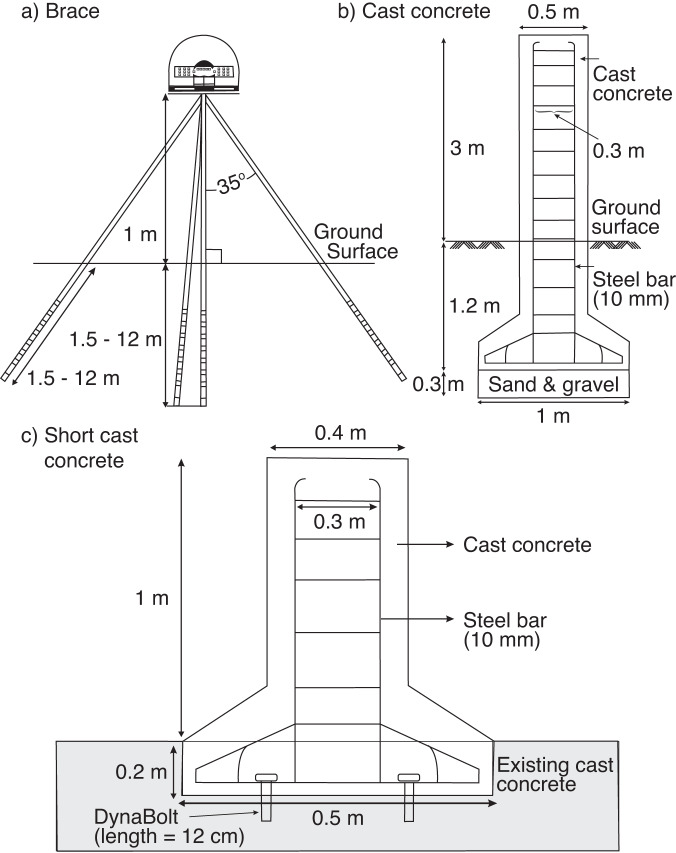
Monument types of the BIG GNSS stations used in this study.

**Fig. 3:**
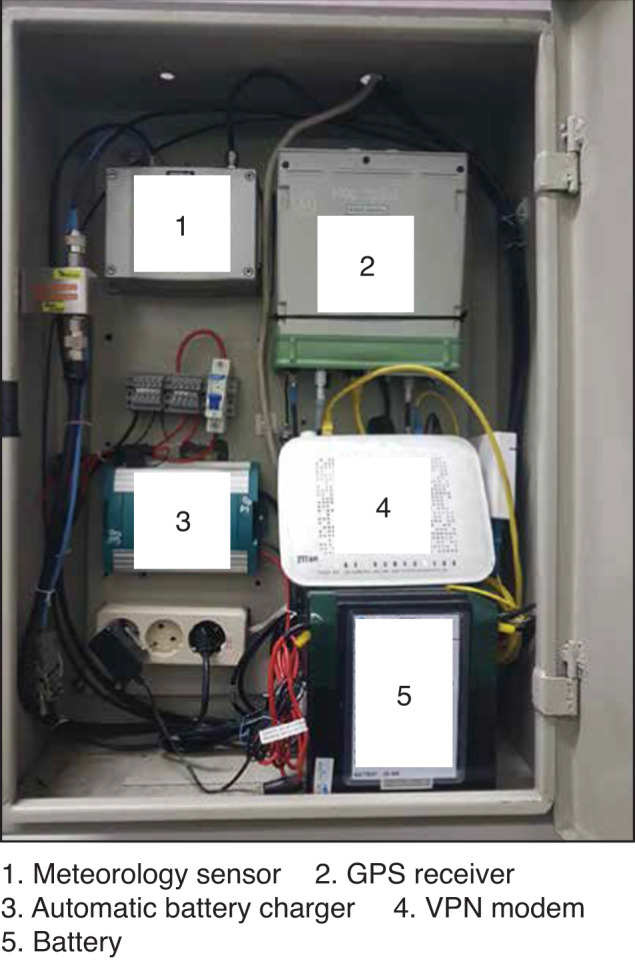
Components of the BIG GNSS stations.

We obtain the Receiver Independent Exchange (RINEX) GNSS data from the Geospatial Information Agency of Indonesia (BIG) which has been establishing and maintaining continuous GNSS stations in the country since 1996. Most stations are located on the national telecommunication company network. The stations use different monument types (Fig. 2) and record data continuously at one sample per second using high-precision L1/L2 geodetic type receivers and standard Choke Ring antennas (Table 1). In addition, the stations also have meteorological instrument systems, an automatic battery charger that connects to the national power network, and a cell modem (Fig. 3) that will stream the recorded raw data via a secure TCP/IP connection to BIG’s data processing centre in Cibinong, West Java up to one-hour latency.

**Table 1:** GNSS station specifications.

Site	Monument type	Receiver type	Antenna type	Data transmission	First observation
CPSR	BRACE	TRIMBLE ALLOY	LEIAT504	VPN	2010
CGON	SCC	TRIMBLE ALLOY	LEIAR20	VPN	2010
CTGR	SCC	TRIMBLE ALLOY	LEIAR25	VPN	2009
CJKT	SCC	TRIMBLE ALLOY	LEIAR20	VPN	2010
CBTU	SCC	LEICA GR10	LEIAR25	VPN	2010
CROL	CC	TRIMBLE ALLOY	LEIAR25	VPN	2010
CCIR	SCC	LEICA GR50	TPSCR.G3	VPN	2010
CTGL	SCC	LEICA GR50	LEIAR25	VPN	2010
CSEM	SCC	LEICA GR50	TPSCR.G3	VPN	2010
CJPR	CC	TRIMBLE ALLOY	HX-C6X601A	VPN	2010
CPKL	CC	LEICA GR50	LEIAR25	VPN	2010
CPWD	SCC	LEICA GR50	LEIAR20	VPN	2010
CTBN	CC	TPS NET-G3A	TPSCR.G3	VPN	2010
CLMG	SCC	LEICA GR50	TPSCR.G3	VPN	2010
CMJT	SCC	LEICA GR50	TPSCR.G3	VPN	2010
CSBY	SCC	LEICA GR50	TPSCR.G3	VPN	2010
CPAS	SCC	TPS NET-G3A	TPSCR.G3	VPN	2010
CPAI	SCC	TPS NET-G3A	TPSCR.G3	VPN	2010
CSIT	SCC	TPS NET-G3A	TPSCR.G3	VPN	2010
CBRN	BRACE	TRIMBLE ALLOY	LEIAT504	Offline	2008

CC: Cast Concrete; SCC: Short Cast Concrete.

## Methods

We processed the RINEX GNSS data and obtained a time series of GNSS station coordinates using the GPS at MIT/Global Kalman filtering (GAMIT/GLOBK) software package version 10.71^45–47^. Our GPS processing consisted of two steps^48,49^. In the first step, we used double-differencing methods in the GAMIT software to estimate daily station positions, atmospheric parameters, satellite orbits, and earth orientation parameters from ionosphere-free linear combination GPS phase observations. During this step, we fixed the satellite orbit parameters to the IGS final orbits and applied a second-order ionospheric correction using IGS final ionospheric products. We set the computation parameters to the default GAMIT setting, except for the atmospheric delay parameters, which were modeled and estimated every hour using the Vienna Mapping Function^50^. We corrected the station displacements due to ocean tides using the most recent global ocean tide model, Finite Element Solution 2004^51^. To adjust the effect of solar and solid-earth tides, we applied the International Earth Rotation and Reference System Service 2010 standard model^52^ and the atmospheric pressure loading model corrections^53^. Finally, we included GPS data from 12 International GNSS Services (IGS) stations (ALIC, BAKO, COCO, DARW, DGAR, GUAM, HYDE, IISC, LHAZ, PIMO, XMIS, YARR) in our daily processing to integrate our local network into the ITRF2014 reference frame^54^.

In the second step, we used the GLOBK software to combine our daily solutions with the global GPS solutions provided by the MIT analysis centre. During this step, we aligned our combined solutions with the ITRF2014 reference frame^54^ by minimising the position differences of eight selected sites^55^, using a priori values defined by the IGb14 realisation of ITRF2014^54^. To accomplish this position difference minimation, we calculated six Helmert transformation parameters (three translations and three rotations) of eight selected reference sites: YARR in Australia, MAW1 and DAV1 in Antarctica, STJO and FLIN in North America, WSRT, ONSA, and NOT1 in Europe^55^. These sites were selected because they are less affected by earthquake deformations and hydrological loading^55^. Lastly, we generated daily time series coordinates for all the GNSS stations with respect to IGb14 realisation of ITRF2014^54^.

## Data Records

**Fig. 4:**
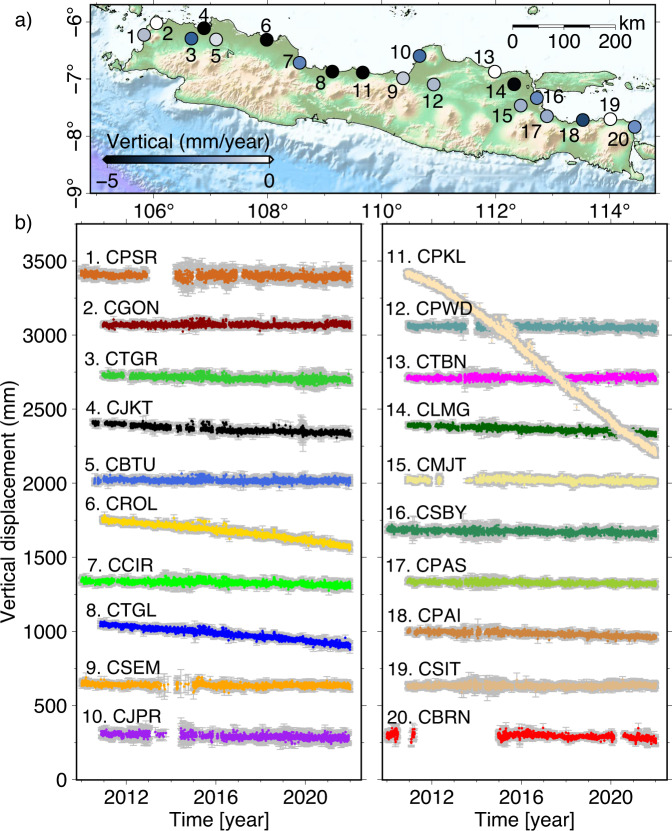
Vertical component of the twenty BIG GNSS stations. (**a**) Negative vertical velocities are likely related to land subsidence. (**b**) Daily time series of the GNSS vertical component from 2010 to 2021.

The processing results are a time series of 3D displacements from 2010 to 2021, relative to the ITRF2014. Most stations record negative velocities in the vertical component and are likely related to land subsidence (Table 2 and Fig. 4). The time series of the 3D displacements that include horizontal motions can be found in this repository: 10.5281/zenodo.7775016^56^.

**Table 2:** Vertical velocity recorded by the BIG GNSS stations along the northern coastline regions of Java.

Site	Longitude (degree)	Latitude (degree)	Vertical velocity (mm/year)	Uncertainty (mm/year)
CPSR	105.834	−6.226	−1.0	0.052
CGON	106.052	−6.021	0.0	0.043
CTGR	106.664	−6.291	−2.9	0.049
CJKT	106.885	−6.110	−6.4	0.043
CBTU	107.096	−6.308	−0.5	0.044
CROL	107.985	−6.313	−15.9	0.045
CCIR	108.561	−6.716	−2.3	0.041
CTGL	109.136	−6.871	−12.5	0.043
CSEM	110.377	−6.987	−0.8	0.038
CJPR	110.667	−6.596	−2.7	0.056
CPKL	109.669	−6.887	−107.0	0.202
CPWD	110.914	−7.096	−1.1	0.044
CTBN	111.986	−6.872	0.4	0.042
CLMG	112.327	−7.093	−4.9	0.041
CMJT	112.442	−7.466	−1.3	0.042
CSBY	112.724	−7.334	−2.2	0.039
CPAS	112.901	−7.651	−1.4	0.033
CPAI	113.530	−7.719	−3.5	0.039
CSIT	114.013	−7.703	0.1	0.041
CBRN	114.440	−7.838	−2.1	0.082

## Technical Validation

Bad environments (e.g., buildings and trees) that degrade the sky view of the GNSS antenna will reflect and refract satellite signals before arriving at the antenna^57^. The reflected and refracted signals are called multipath signals which will introduce carrier phase measurement errors and subsequently lead to a positioning error^58^. A simple approach to inspect the environments surrounding the antenna is by plotting the signal-to-noise ratio (SNR) values measured by the GNSS receivers^59^. The SNR, like the carrier phase measurement, is also impacted directly by multipath signals and hence can therefore be used as a proxy to assess the environments surrounding the GNSS antenna^59,60^. Low SNR values indicate a large tracking error^59^, meaning that multipath objects are present. We plot the SNR values using the L1 data recordings only. We do not use the L2 data due to its encrypted C/A code and the lack of civilian access to the P-code which affects the L2 SNR reliability^59^. We plot the SNR values as a function of azimuth and elevation angle, both in the time series and sky plot (Figure S1). The SNR plot shows that all the stations have SNR values greater than 30 decibels, indicating good environments surrounding the GNSS stations hence the negative velocities in the vertical component are robust.

**Fig. 5:**
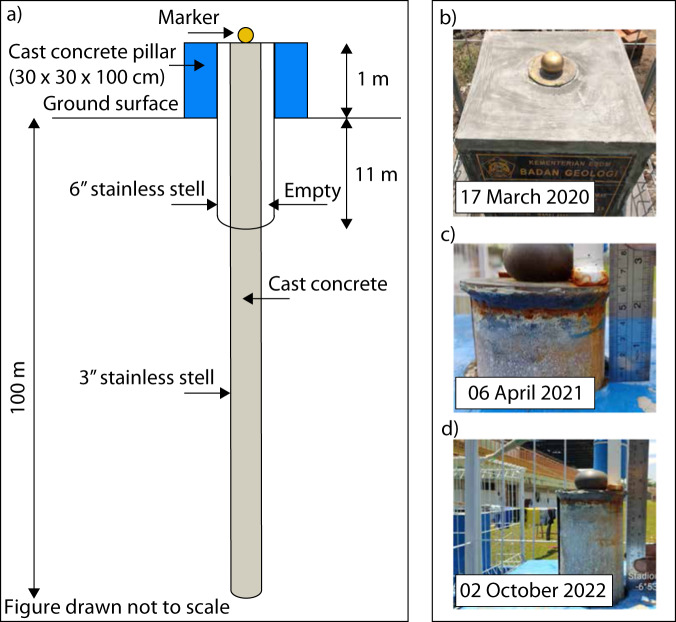
A deep pile benchmark to measure land subsidence in Pekalongan. (**a**) A schematic design of the deep pile benchmark. (**b**) The newly installed benchmark. (**c**) The benchmark measures 60 ± 1 mm land subsidence by 06 April 2021. (**d**) The benchmark measures 140 ± 1 mm land subsidence by 02 October 2022, meaning that 80 ± 1 mm land subsidence occurs within ~1.5 years.

In addition to SNR analysis, we use independent observation measured by a deep pile benchmark to validate the negative velocity at the GNSS CPKL station in Pekalongan (Table 2 and Fig. 4). The benchmark (Fig. 5) was installed by the centre for groundwater and environmental geology, Indonesia’s geological agency, on 17 March 2020 ~500 m northeast of the GNSS CPKL station. The benchmark measurements between 06 April 2021 and 02 October 2022 estimate that ~80 ± 1 mm land subsidence occurs within ~1.5 years (Fig. 5c,d). Unfortunately, we cannot make a one-to-one comparison between this result and the amplitude of land subsidence measured at the GNSS CPKL station due to two reasons: 1) the benchmark and the GNSS CPKL station are ~500 m apart, 2) our GNSS data ended in 2021. Nevertheless, the benchmark measurements are still important in the sense that Pekalongan city is experiencing severe land subsidence.

## Supplementary information

GNSS land subsidence observations along the northern coastline of Java, Indonesia

## Data Availability

The GAMIT/GLOBK software we used to process the GNSS data is available at http://geoweb.mit.edu/gg/. The scripts we used to do the SNR analysis are available at https://github.com/ericlindsey/gnss-snr-skyplot.
