# Supplementary material for: GNSS land subsidence observations along the northern coastline of Java, Indonesia
Source: Sci Data. 2023 Jul 1;10:421. doi: 10.1038/s41597-023-02274-0 (PMC10314896; doi:10.1038/s41597-023-02274-0)
Supplement: Supplementary file 1 — GNSS land subsidence observations along the northern coastline of Java, Indonesia [file 41597_2023_2274_MOESM1_ESM.pdf]

Supplementary information to

GNSS land subsidence observations along the northern coastline of Java, Indonesia

Susilo Susilo[1], Rino Salman[2], Wawan Hermawan[3], Risna Widyaningrum[3], Sidik Tri Wibowo[4], Yustisi Ardhitasari Lumban-Gaol[1], Irwan Meilano[5], Sang-Ho Yun[2,6,7]

1. National Agency for Research and Innovation (BRIN), Indonesia
2. Earth Observatory of Singapore, Nanyang Technological University, Singapore
3. Center for Groundwater and Environmental Geology, Geological Agency, Indonesia
4. Geospatial Information Agency (BIG), Indonesia
5. Institute of Technology Bandung (ITB), Indonesia
6. Asian School of the Environment, Nanyang Technological University, Singapore
7. School of Electrical and Electronic Engineering, Nanyang Technological University, Singapore

Corresponding author(s): Susilo Susilo (susilo.2@brin.go.id) and Rino Salman (rino@ntu.edu.sg)

Table of Contents

Figure S1. SNR plot of all the GNSS stations based on the L1 data recordings. a) Time series of the SNR values from 2010 to 2021 drawn as multicolour lines based on their azimuth and elevation angles (see CBRN station for the legends). Vertical solid lines mark the times of receiver replacement, vertical dashed lines mark the times of antenna replacement, and dot dashed lines for both. b) SNR values plotted spatially across the azimuth and elevation angles. Azimuth-wise, the circles are divided into six quadrants (60° each). Elevation-wise, the circles are divided into 0–45° (regions within the coloured triangles) and 45–90° (regions within the coloured quadrilaterals). 6

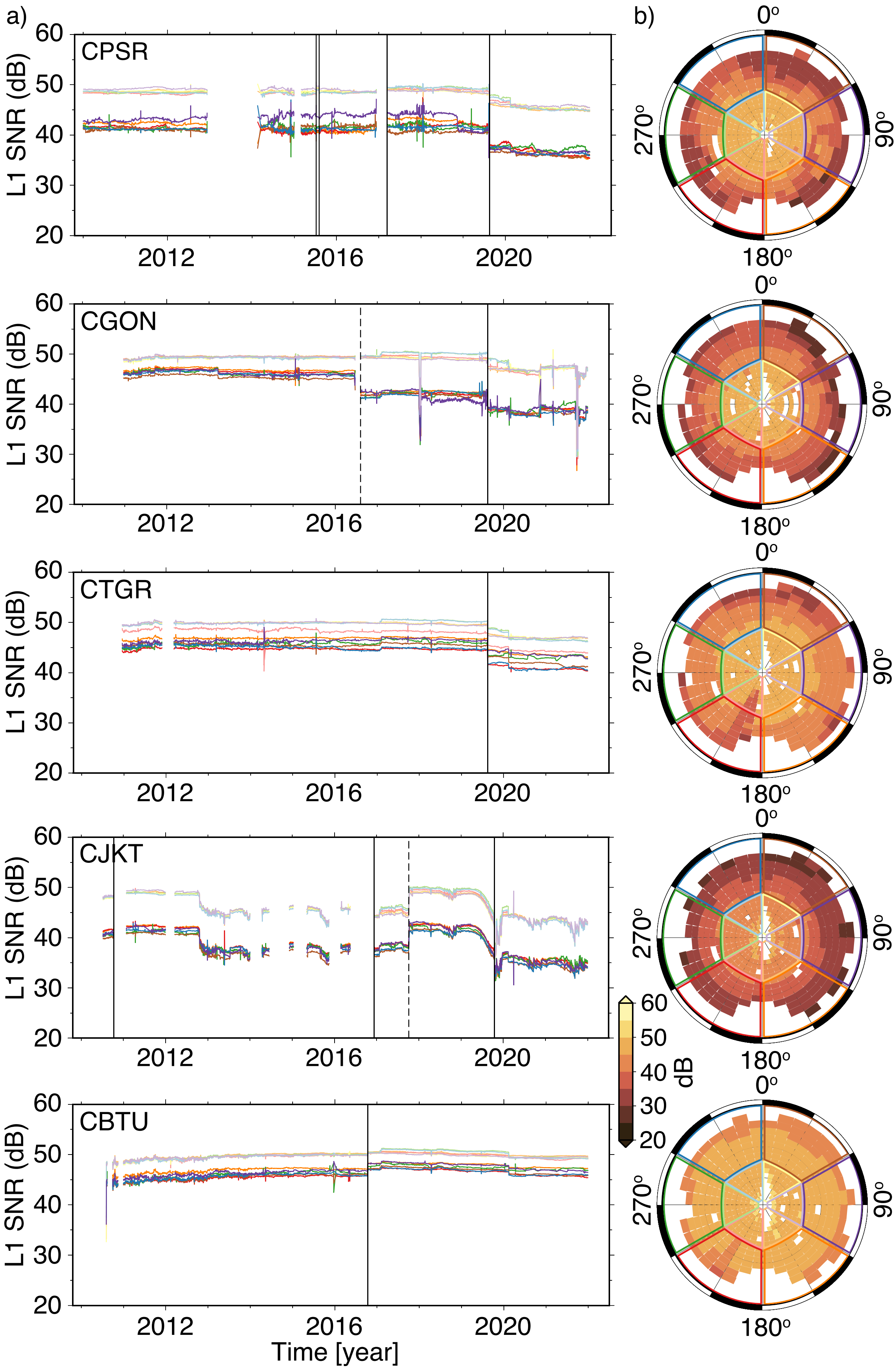

a)
b)
CPSR
CGON
CTGR
CJKT
CBTU
L1 SNR (dB)
Time [year]
2012
2016
2020
60
50
40
30
20
0°
90°
180°
270°
dB

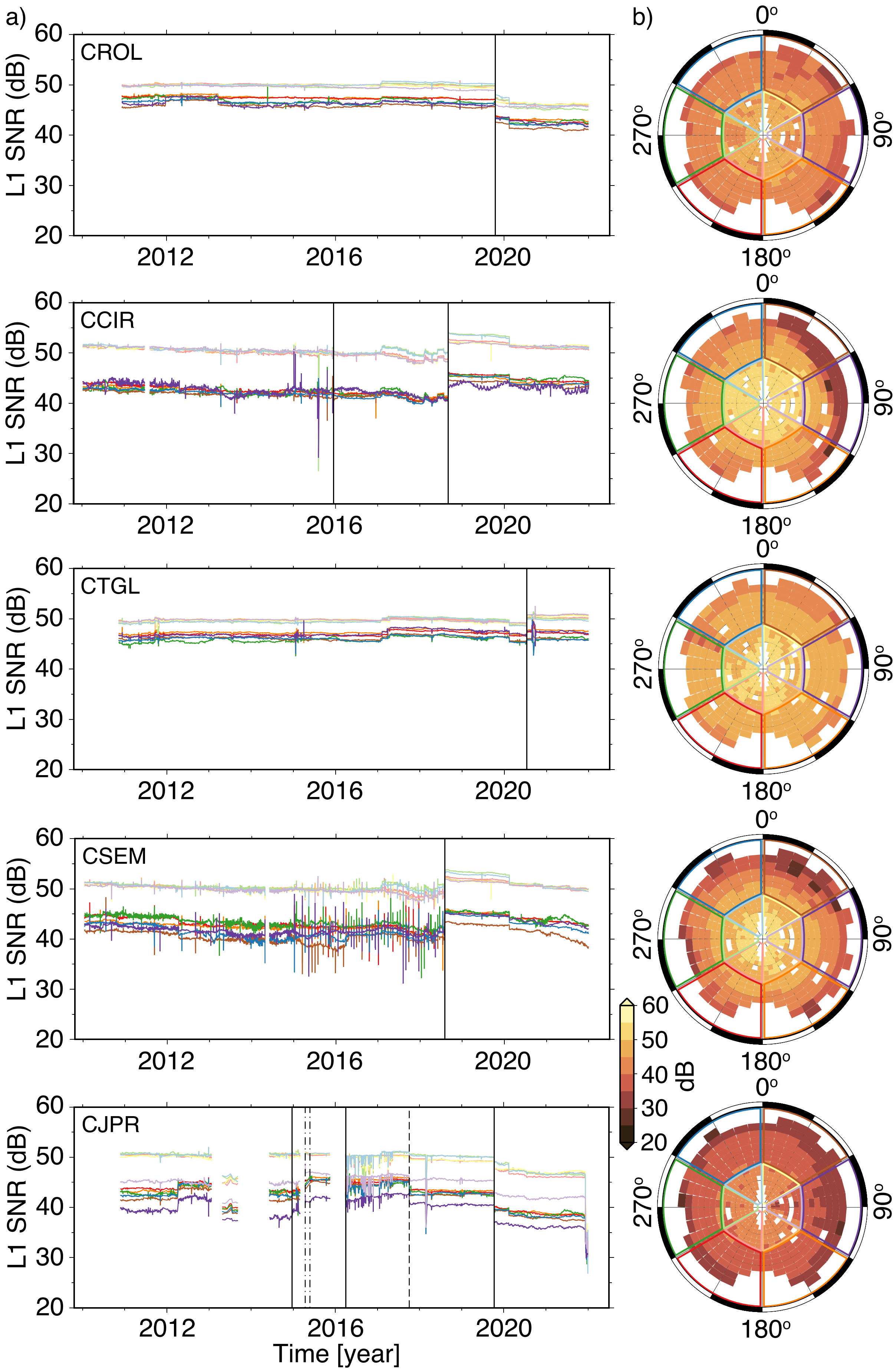
a)
b)
CROL
CCIR
CTGL
CSEM
CJPR
L1 SNR (dB)
60
50
40
30
20
2012
2016
2020
Time [year]
0°
90°
180°
270°
60
50
40
30
20
dB

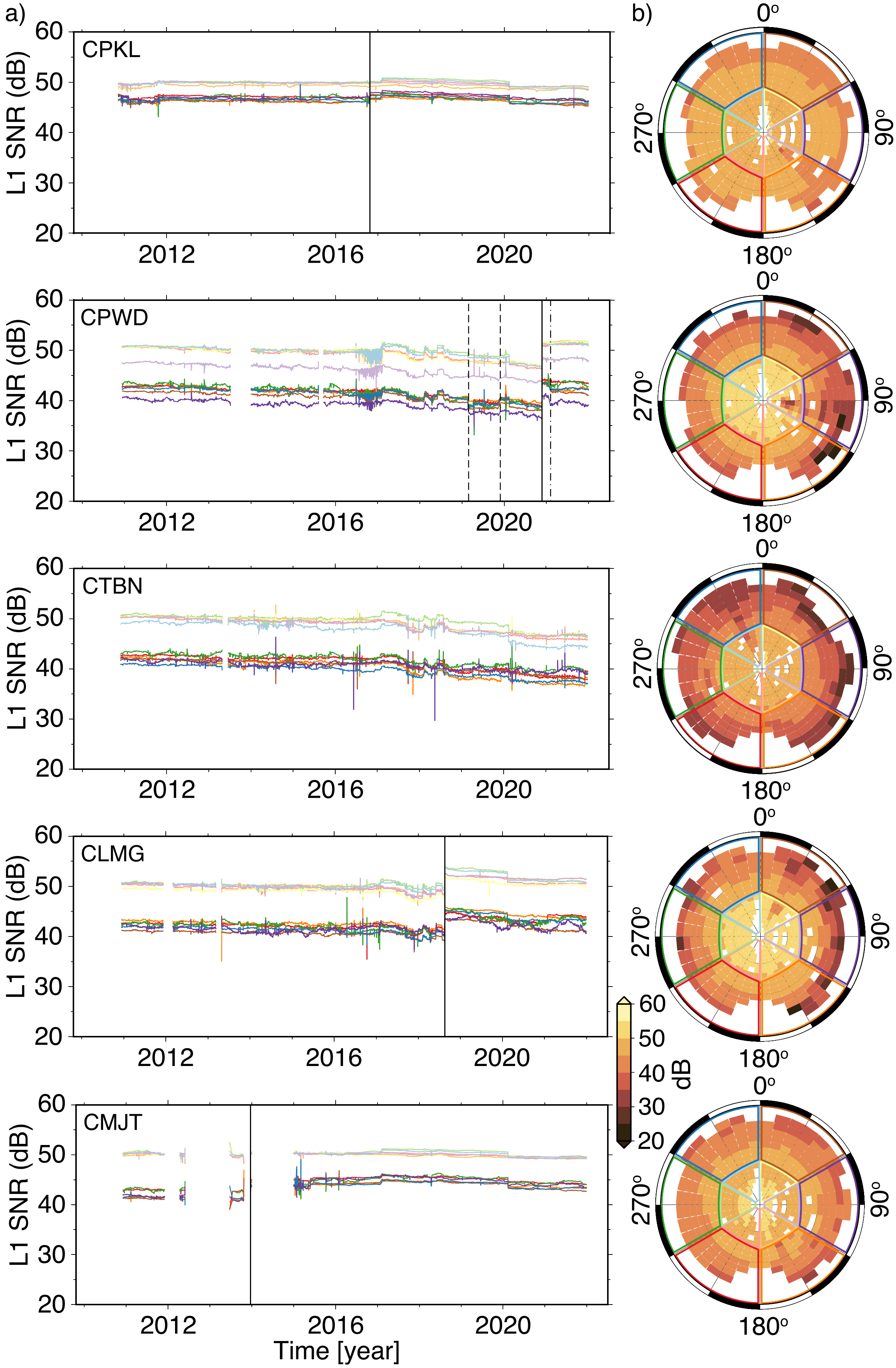

a)
b)
CPKL
CPWD
CTBN
CLMG
CMJT
L1 SNR (dB)
60
50
40
30
20
2012
2016
2020
Time [year]
0°
90°
180°
270°
dB

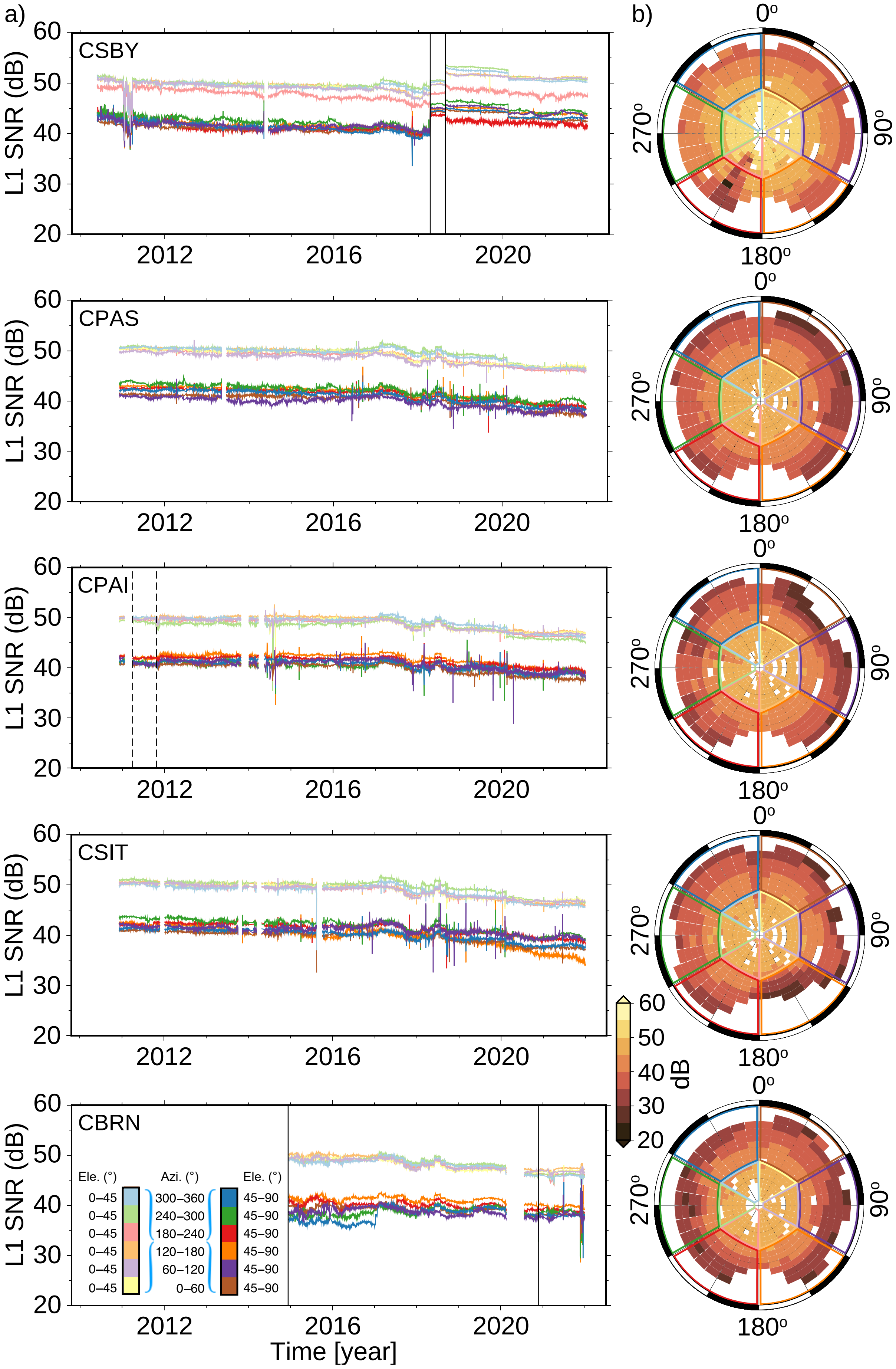
a)
b)
CSBY
CPAS
CPAI
CSIT
CBRN
L1 SNR (dB)
Time [year]
2012
2016
2020
0°
90°
180°
270°
dB
Ele. (°)
Azi. (°)
0–45
45–90
300–360
240–300
180–240
120–180
60–120
0–60

Figure S1. SNR plot of all the GNSS stations based on the L1 data recordings. a) Time series of the SNR values from 2010 to 2021 drawn as multicolour lines based on their azimuth and elevation angles (see CBRN station for the legends). Vertical solid lines mark the times of receiver replacement, vertical dashed lines mark the times of antenna replacement, and dot dashed lines for both. b) SNR values plotted spatially across the azimuth and elevation angles. Azimuth-wise, the circles are divided into six quadrants (60° each). Elevation-wise, the circles are divided into 0–45° (regions within the coloured triangles) and 45–90° (regions within the coloured quadrilaterals).
